# Metabolomics-Based Discovery of Diagnostic Biomarkers for Onchocerciasis

**DOI:** 10.1371/journal.pntd.0000834

**Published:** 2010-10-05

**Authors:** Judith R. Denery, Ashlee A. K. Nunes, Mark S. Hixon, Tobin J. Dickerson, Kim D. Janda

**Affiliations:** 1 Department of Chemistry and Worm Institute for Research and Medicine, The Scripps Research Institute, La Jolla, California, United States of America; 2 Department of Immunology and Microbial Science, The Scripps Research Institute, La Jolla, California, United States of America; University of Oklahoma Health Sciences Center, United States of America

## Abstract

**Background:**

Development of robust, sensitive, and reproducible diagnostic tests for understanding the epidemiology of neglected tropical diseases is an integral aspect of the success of worldwide control and elimination programs. In the treatment of onchocerciasis, clinical diagnostics that can function in an elimination scenario are non-existent and desperately needed. Due to its sensitivity and quantitative reproducibility, liquid chromatography-mass spectrometry (LC-MS) based metabolomics is a powerful approach to this problem.

**Methodology/Principal Findings:**

Analysis of an African sample set comprised of 73 serum and plasma samples revealed a set of 14 biomarkers that showed excellent discrimination between *Onchocerca volvulus*–positive and negative individuals by multivariate statistical analysis. Application of this biomarker set to an additional sample set from onchocerciasis endemic areas where long-term ivermectin treatment has been successful revealed that the biomarker set may also distinguish individuals with worms of compromised viability from those with active infection. Machine learning extended the utility of the biomarker set from a complex multivariate analysis to a binary format applicable for adaptation to a field-based diagnostic, validating the use of complex data mining tools applied to infectious disease biomarker discovery and diagnostic development.

**Conclusions/Significance:**

An LC-MS metabolomics-based diagnostic has the potential to monitor the progression of onchocerciasis in both endemic and non-endemic geographic areas, as well as provide an essential tool to multinational programs in the ongoing fight against this neglected tropical disease. Ultimately this technology can be expanded for the diagnosis of other filarial and/or neglected tropical diseases.

## Author Summary

Onchocerciasis, caused by the filarial parasite *Onchocerca volvulus*, afflicts millions of people, causing such debilitating symptoms as blindness and acute dermatitis. There are no accurate, sensitive means of diagnosing *O. volvulus* infection. Clinical diagnostics are desperately needed in order to achieve the goals of controlling and eliminating onchocerciasis and neglected tropical diseases in general. In this study, a metabolomics approach is introduced for the discovery of small molecule biomarkers that can be used to diagnose *O. volvulus* infection. Blood samples from *O. volvulus* infected and uninfected individuals from different geographic regions were compared using liquid chromatography separation and mass spectrometry identification. Thousands of chromatographic mass features were statistically compared to discover 14 mass features that were significantly different between infected and uninfected individuals. Multivariate statistical analysis and machine learning algorithms demonstrated how these biomarkers could be used to differentiate between infected and uninfected individuals and indicate that the diagnostic may even be sensitive enough to assess the viability of worms. This study suggests a future potential of these biomarkers for use in a field-based onchocerciasis diagnostic and how such an approach could be expanded for the development of diagnostics for other neglected tropical diseases.

## Introduction

Onchocerciasis, commonly referred to as “river blindness” is classified by the World Health Organization (WHO) as a neglected tropical disease, afflicting approximately 37 million people in Africa, Central and South America and Yemen, with 89 million more at risk [1]. Symptoms of the disease include acute dermatitis and blindness, the result of which is the loss of 1 million disability-adjusted life years (DALYs) annually [2]. The causative agent, the filarial nematode *Onchocerca volvulus*, is transmitted in its larval stage between human hosts through the bite of a *Simulium* (sp.) black fly. Once these parasites have matured into the adult form, they can live for approximately 14 years in subcutaneous nodules within a human host [3]. The drug ivermectin (Mectizan) has served as the principal means of onchocerciasis control [4], however, after initially reducing the number of microfilariae, within a year, the microfilariae return to levels of 20% or higher than that prior to treatment [5]. The combination of the lack of effect of annual ivermectin treatment on adult worm survival and the fecundity of adult females, along with significant fly and human migration patterns has helped to perpetuate the disease.

In Africa, where onchocerciasis control programs have been in place since the founding of the Onchocerciasis Control Programme in West Africa (OCP, 1974–2002) and are currently being conducted by the African Programme for Onchocerciasis Control (APOC, 1995-present), diagnosis is an essential aspect of the determination of treatment and distribution of medication. In the Western hemisphere, accurate and robust diagnostics are essential for attaining the goal of disease elimination. Twice yearly dosage of ivermectin, through the efforts of the Onchocerciasis Elimination Program for the Americas (OEPA, 1992-present), has lead to a minimization of infection to 13 foci within six countries in Central and South America. Although mass treatment of onchocerciasis foci in the Western hemisphere is slated to be suspended in 2012 [6], achieving the goal of elimination is contingent upon continued surveillance of the disease. However, proper surveillance is directly dependent on the availability of robust diagnostic technologies used for infection assessment. This need is further underscored in studies of antibiotic treatments being investigated for targeting *Wolbachia* endosymbiotic bacteria [7]–[10] as well as reports of sub-optimal response to ivermectin treatment [11], [12]. In both of these cases an accurate diagnostic is critical for the analysis of drug efficacy and patient drug response.

Currently, multinational control and elimination programs primarily rely on various techniques for diagnosis including: entomological studies of *Simulian* flies, *Ov* specific antigen tests, antibody tests, analysis of microfilariae in skin snips, nodule palpation and quality of those nodules that can be excised. There are a number of technical concerns with each technique including: a lack of sensitivity and reproducibility, invasiveness, and the inability to distinguish past from present infection or between filarial diseases [13]–[15]. A small molecule/metabolite based test has the potential for reflecting a more accurate picture of infection status, as it is a comprehensive measure of the effects of posttranslational modification and regulation. Furthermore, small molecules are frequently constitutively produced (e.g., excretory-secretory products), diffuse easily and are inherently non-immunogenic *in vivo*, thus avoiding some of the technical challenges associated with DNA and protein-based diagnostics.

Although adult *O. volvulus* worms do not reside directly in the blood, the highly vascularized subcutaneous nodules of the human host allow for the potential diffusion of adult parasite-derived compounds into the blood where compounds involved in host response to infection might also be present. Since the microfilariae (mf) and third infective larval stage (L3) of the *O. volvulus* life cycle do come in contact with the vascular system during vector transmission, it is additionally possible that some mf or L3 produced compounds might also be localized to this biological sample. Certainly, as a starting point, the blood matrix serves as an easy to obtain, chemically complex data rich matrix for metabolite analysis [16], [17].

However, a technical challenge of analyzing a large number of metabolites stems from the shear size and complexity of the resulting data set. Initially devised and applied to the analysis of highly dimensional gene micro-array data, a number of machine learning approaches have been expanded and used for identifying patterns of biomarkers resulting from the multidimensional analysis of genes, proteins, and metabolites that can be linked to early detection [18], survival prediction [19], and disease outcomes [20]. Although identification of a single biomarker “smoking gun” is perceived as the ideal scenario, more attention is being focused on the use of multiple markers for improving overall diagnostic accuracy [18], [21], [22] and model stability [23].

Herein, we report a liquid chromatography-mass spectrometry (LC-MS) based approach to the discovery of a set of molecules that, in combination, provide a statistically relevant characteristic of onchocerciasis infection. An initial untargeted analysis was applied to the profiling of *O. volvulus* infected and uninfected blood plasma and serum samples representing a variety of geographic regions and disease states, including other tropical diseases. This analysis resulted in a set of statistically significant mass features identified for their potential as onchocerciasis-specific biomarkers. Using multivariate statistics and machine learning algorithms, these metabolic signatures were further evaluated for their ability to discriminate *O. volvulus*-infected and uninfected individuals, therefore, creating the basis of a small molecule-based diagnostic for onchocerciasis.

## Methods

### Ethics statement

The use of human serum and plasma samples in the study was approved by the Scripps Health Human Subjects Committee. Samples with geographic origins outside of the United States of America (USA) consisted of pre-existing, unidentifiable diagnostic specimens collected with written informed consent and in cases of illiteracy, a literate witness signed and a thumbprint was made by the participant. These samples were determined by the Scripps Health institutional review board (IRB) to be exempt from formal review under 45 CFR 46 101. *O. volvulus* negative controls from the USA consisted of serum and plasma samples and were obtained with written informed consent from healthy donors through The Scripps Research Institute Normal Blood Donor Service and approved by the Scripps Health IRB. All patient codes have been removed in this publication.

### Diagnostic sample origin

Onchocerciasis positive samples were collected in characterized endemic areas and their status confirmed by either positive skin snip (mf +) or nodule palpation (nodule +). Several sample groups used in this analysis were collected during previously published studies including serum from Liberia [24], [25] and Ghana collected in 2003 [9]. The Ghana sera collected in 1986 and 1991 were obtained from the College of Public Health, University of South Florida. Cameroon samples were obtained as part of a nodulectomy campaign conducted in villages surrounding Kumba, Cameroon in 2006 and consist of plasma from *O. volvulus*-positive individuals (nodule + with nodules containing live females), *O. volvulus*-negative individuals (skin snip - volunteers with no current or prior symptoms of *O. volvulus* infection), and ambiguous samples (nodules contained either dead, calcified worms or lipomas with no evidence of worms, or for which there were no particular disease symptoms recorded). Guatemala sera were obtained as part of a nodulectomy campaign conducted by the Guatemala Ministry of Health and the Centro de Estudios en Salud, Universidad del Valle de Guatemala in several villages within the Guatemalan Central Endemic Zone from 2007–2008. Nodules were surgically removed from all individuals sampled, and nodule dissection was conducted to assess worm viability on nodules from five of the 21 individuals whose serum was analyzed in this study. Of those dissected, no live worms were found. Leishmaniasis positive, Chagas disease positive, and onchocerciasis negative sera were obtained from the Centro de Estudios en Salud, Universidad del Valle de Guatemala. Indian lymphatic filariasis positive plasma samples were obtained from the Laboratory of Parasitic Diseases, U.S. National Institutes of Health. A detailed summary of the samples analyzed in this study is presented in Table 1.

**Table 1:** Summary of samples analyzed in this study.

Country of origin	Matrix	Number/description	Clinical pathology	Nodulectomy results	Average mf per mg
Cameroon	Plasma	16 *O. volvulus* infected	Palpable nodule(s)	All nodules contained live worms	—a
		18 uninfected controls	—	NA	0^b^
		1 calcified worm	Palpable nodule(s)	1 dead, calcified worm	—
		2 ambiguous lipoma	Palpable nodule(s)	Fat deposit	—
		1 indeterminant infection status	—	—	—
Ghana	Serum	15 *O. volvulus* infected	Palpable nodule(s)	—	12^c^
		10 *O.volvulus*, mf+	Acute papular onchodermatitis	—	+^d^
Liberia	Serum	10 *O. volvulus* infected	—	—	527^e^
Guatemala	Serum	21 *O. volvulus* infected	Palpable nodule(s)	24% of nodules sampled all worms dead	—
		17 uninfected controls	—	NA	—
		6 *Leishmania braziliensis*	—	NA	—
		6 *Leishmania mexicana*	—	NA	—
		5 *Trypanosoma cruzi*	—	NA	—
India	Plasma	4 *Wuchereria bancrofti*	—	NA	—
USA	Plasma	3 *O.volvulus* uninfected, no known disease	—	NA	—
	Serum	3 *O. volvulus* uninfected, no known disease	—	NA	—

a — indicates no measurement made.

b no mf were measured in samples collected from four different anatomical locations from the 18 control individuals included in this analysis.

c Average number of mf measured per patient in original study [9] using two skin snips [49].

d Samples are known to be mf+, but records are not available.

e Average number of mf collected from six different anatomical locations from the 10 patients included in this analysis.

### Sample preparation and metabolite extraction

Solvents used were of high performance (HPLC) grade. A methanol precipitation of proteins was conducted by adding 400 µl aliquots of ice cold methanol to 100 µl aliquots of serum and plasma samples. The samples were immediately vortexed for 30 sec and allowed to rest on ice for 20 min. After centrifugation at 13,780×g for 5 min, the metabolite containing supernatent was removed from the precipitated protein pellet and transferred to fresh tubes. The supernatent samples were dried in a GeneVac EX-2 Evaporation System (GeneVac Inc., Valley Center, New York, USA) at ambient temperature and then resuspended to a 50 µl volume in water: acetonitrile (95∶5), vortexed for 30 sec and then centrifuged again at 13,780×g for 5 min. After being transferred to LC vials, samples were stored at 4°C and transferred to the LC-MS thermostated autosampler (6°C), typically within 48 h of their preparation.

### Chromatographic workflow

In order to minimize instrumental drift, sample sequences were composed of a single injection of each sample in randomized order. To monitor any potential instrument irreproducibility and to confirm the absence of sample carry over within the chromatographic run, a mobile phase blank and an external standard were injected every 24 h throughout the duration of the analysis.

### LC-ESI MS analysis

Experiments were performed with an electrospray-ionization time-of-flight (ESI-TOF) MS (Agilent 1200 LC, TOF 6210, Agilent Technologies, Santa Clara, CA, USA). Each sample analysis consisted of an 8 µl injection of extracted sample with chromatographic separation across a reverse phase C18 column (Zorbax 300SB C18 Capillary, 3.5 µm, 1 mm×150 mm; Agilent Technologies, Santa Clara, CA, USA) at a capillary pump flow rate of 75 µl/min. Mobile phase A was composed of water with 0.1% formic acid, and mobile phase B was acetonitrile with 0.1% formic acid. Each sample was analyzed over a 60 min run time with a gradient consisting of a 45 min linear gradient from 5% to 95% B and 15 min isocratic hold at 95% B. Between sample injections a wash step was used to minimize carry over. It consisted of a saw-tooth linear gradient beginning with a hold at 95% A for 10 min. Then, linear ramping between 5% and 98% B for 5 minute increments throughout the 35 min wash cycle was followed by a 20 minute final re-equilibration of the column with an isocratic hold at 95% A.

### Mass spectrometric conditions

Consistent mass accuracy (<2 ppm) was maintained through the constant infusion (2 µl/min) of reference masses via a second nebulizer. Data were collected in positive electrospray ionization (ESI) mode scanning in centroid mode from 75 to 1,100 *m*/*z* with a scan rate of 1.0 spectrum per second in 2 GHz extended dynamic range. The capillary voltage was 3,500 V; the nebulizer pressure, drying gas flow and gas temperature were set to 20 psig, 12 l/min and 350°C, respectively.

### Data pre-processing, pattern determination, and statistical analysis

All mass spectral data was collected in .d format and converted to .mzData using the Mass Hunter Qualitative Analysis software version B.03.01 (Agilent Technologies, Santa Clara, CA, USA). XCMS [26] software was used for peak matching, non-linear retention time alignment and quantitation of mass spectral ion intensities across all .mzData mass spectral files. Statistical comparison of the intensity data was conducted using the XCMS built in Welch's *t*-test. False discovery rate (FDR) analysis was conducted with the q-value program [27] in R version 2.9.0 [28]. Principal Components Analysis (PCA) was conducted with Statistica software version 8.0 (StatSoftInc., Tulsa, OK, USA), machine learning algorithms were implemented using Weka Explorer version 3.6.0 [29] with 10 fold cross-validation settings.

### Compound formula assignment

The molecular formula assignment made for the 10 selected small molecule biomarkers was conducted through a combination of LC-MS/MS fragmentation using a quadrupole- TOF MS (QTOF 6510, Agilent Technologies, Santa Clara, CA, USA) and sub-2 ppm accurate mass measurements using a Bruker Daltonics Apex II 7.0 Tesla Fourier transform ion cyclotron (FT-ICR) MS (Bruker Daltonics., Billerica, MA,USA). For the QTOF analysis chromatographic conditions were identical to those reported for the profiling experiment and serum plasma samples from either the Scripps normal blood or pooled patient samples were used for the analysis. The average *m/z* and retention times of each of the biomarkers obtained through XCMS analysis, were used for targeted MS/MS analysis with a starting collision-induced dissociation energy of 20eV. Fragmentation patterns were analyzed with the Agilent Mass Hunter Qualitative Analysis software version B.03.01 using the targeted MS/MS and formula generation algorithms and compared with the MS/MS fragment data in the METLIN database [30].

The FTMS system was equipped with a custom machined electrospray source with two nebulizers for dual spray ionization. The main orthogonal nebulizer was used for LC-eluent, while the second nebulizer was used to introduce a calibration mixture containing two compounds (aminoantipyrine at 204.1132 *m/z* and quinidine 325.1911 *m/z*) at 3 mM concentration mixed with 1∶10 dilution of Agilent low concentration tune mix. A linear calibration fit was used in the narrow range to internally calibrate individual mass spectra. The chromatographic conditions were identical to those reported for the profiling experiment with an additional analysis using a smaller i.d. column with the same stationary phase composition (Zorbax 300SB C18 Capillary, 3.5 µm, 0.3 mm×150 mm; Agilent Technologies, Santa Clara, CA, USA) at a capillary pump flow rate of 4µl/min. Pooled serum and plasma samples from either the Scripps normal blood or patient samples were used for the analysis.

## Results

A metabolomic approach was developed to address the need for improvement of diagnostics in onchocerciasis detection. Profiling of blood biomarkers is much less invasive than skin snipping or nodulectomy, and should have the added advantage of increased sensitivity. Antigen tests have been attempted in the past, however, the immunogenicity of the proteins has been a consistent deterrent [31], [32]. An advantage of profiling low molecular weight compounds is that they are typically not immunogenic (i.e., M.W. <1,100 amu) and therefore not subject to such a limitation. It is important to note that profiling molecules with molecular weight less than 1,100 amu will also include peptides and/or protein fragments, expanding the pool of available analytes that can be detected.

### Mass spectrometric biomarker selection

The most important aspect of any clinical analytical study resides with the quality of the samples used; here representative serum and plasma samples from a variety of subject populations were incorporated to minimize the effects of non-relevant metabolic variation (e.g., nutrition, sex, age, race) and magnify those metabolic differences that are not only statistically significant between specific populations, but relevant in identifying the changes in metabolism that can be directly attributable to infection.

**Figure 1:**
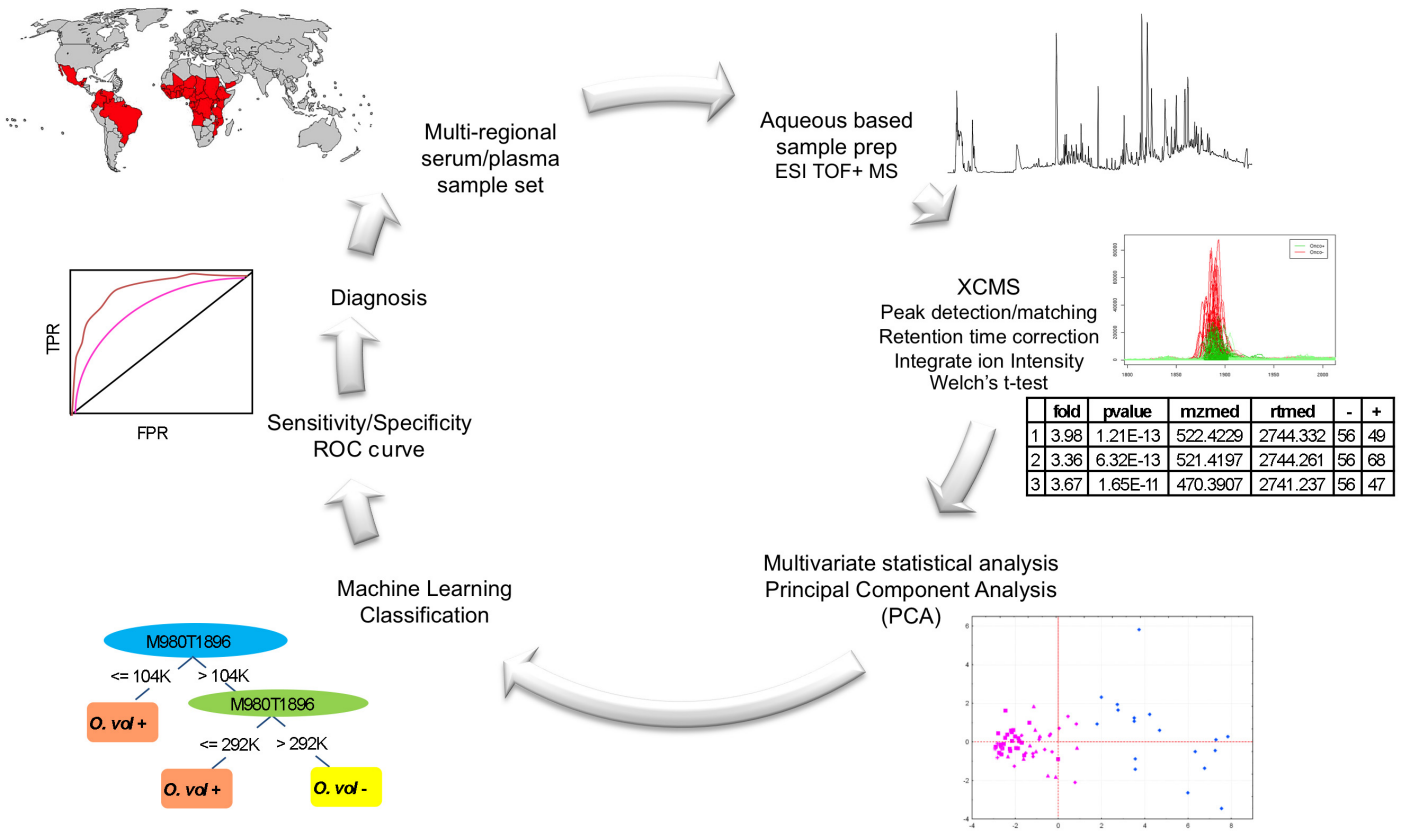
Schematic diagram of the LC-MS based metabolomic workflow. Wherein the multi-region serum and plasma samples are extracted and analyzed within a single sequence on the ESI-TOF in positive mode. Mass spectral data is preprocessed with XCMS software and multivariate statistical analysis and machine learning classification algorithms are used to distinguish patterns in the data and provide a binary output to the classification of samples. ROC curves are used to quantify the relationship between sensitivity and specificity for a given test. Ultimately, this information can be used in an iterative fashion to interrogate larger datasets and provide necessary diagnostic information to better characterize the disease status of clinical samples from a variety of geographic regions.

One of the analytical limitations with an untargeted LC-MS metabolomics approach is that of inter-sequence reproducibility (i.e., sample preparation, instrument drift, column and mass spectral baseline variation) when comparing samples directly between analytical sequences. Such inter-sequence variability can introduce shifts in ion intensities that can interfere with the accuracy of downstream statistical analysis. Therefore, this study was conducted with single injections of each sample, analyzed in randomized order consecutively within one analytical sequence (Figure 1). Due to such analytical constraints, small groups of representative samples were selected from various sample classes (e.g., *O. volvulus*-infected and uninfected individuals from various geographic regions and individuals infected with other parasitic diseases). XCMS analysis of the sample mass spectral data files (n = 136) resulted in the measurement of a total of 2,350 mass features. Testing the overall reproducibility of the analysis, the coefficient of variation (CV) was found to be 15.9% as calculated from all mass feature intensity values compared across triplicate injections of a single plasma sample analyzed throughout the analytical sequence. This value is comparable to previous studies of analytical variation within plasma and serum analysis by our laboratory and consistent with a number of other LC-MS based metabolomics studies [33], [34]. Statistical comparison between all onchocerciasis positive samples (n = 76) and all onchocerciasis negative samples (n = 56), including those infected with other tropical diseases, by Welch's *t*-test resulted in 194 features with a *p*<1×10^−4^; with a false discovery rate FDR of 54%. To reduce the number of potentially erroneous markers and focus on those mass features with the most potential in distinguishing disease, the top 35 mass features (*p*<1×10^−7^) were chosen for more stringent analysis through assessment of the quality of the resulting extracted ion chromatograms (EICs) (Figure S1). While XCMS pre-processing software contains a robust retention time correction and peak alignment algorithm, an important aspect of this study is the statistical quantitation of biomarkers, therefore any features with questionable quantitation, observed as imperfect alignment or inconsistent peak boundaries across samples were ruled out of further analysis. Additionally, since several mass features may redundantly describe one chemical metabolite due to the presence of in-source fragments, adducts, or multiply charged species and overlapping retention time. The features were separated into unique peak groups and representative ions with the highest overall abundance were included in a subset of 14 features for further analysis (Table 2). Interestingly, the majority of these features were detected at lower levels in infected individuals relative to those without onchocerciasis. Analysis of the selected biomarkers with MS/MS and FTMS analysis has provided molecular masses and assigned molecular formulas that could be used to classify the biomarkers into distinct chemical classes; of the 14 markers identified 10 were small molecules and four were protein fragments or small peptides.

**Table 2:** Characteristics of the 14 candidate biomarkers.

Compound Classification	p-value	RT (min)	XCMS average *m/z*	Fold change	Molecular formula	MS/MS major fragments (% abundance)^a^	FTMS accurate mass
Fatty acid/Sterol lipid	6.32×10^−13^	45.7	521.4197	−3.36	C_32_H_56_O_5_	111.0451(39.15)503.4123(29.7)	521.4190
Fatty acid/Sterol lipid	2.06×10^−11^	45.7	469.3872	−3.71	C_28_H_52_O_5_	415.357(100)291.2331(48.57)	469.3888
Sterol lipid	2.16×10^−11^	41.4	425.3611	−3.55	C_26_H_48_O_4_	389.3432(100.0)139.1107(12.8)	425.3625
Protein	3.59×10^−10^	31.6	979.9368	−3.99	-		
Protein	6.53×10^−10^	31.5	986.2677	−5.65	-		
Hexacosenoic acid	4.01×10^−10^	50.7	395.3867	−2.77	C_26_H_50_O_2_	71.0859(100.0)57.0709(81.99)	395.3804
Pentacosenoic acid	7.75×10^−10^	49.1	381.3710	−2.43	C_25_H_48_O_2_	71.0858(100.0)57.0719(58.75)	381.3728
Fatty alcohol/aldehyde	2.39×10^−8^	48.5	241.2505	1.54	C_16_H_32_O	55.0551(77.61)83.0877(46.81)	—b
Fatty acid	1.40×10^−9^	39.0	367.2840	−2.28	C_22_H_38_O_4_	331.2649(100.0)79.0547(33.89)	367.2828
Hydroxy-octadecenoic acid	1.52×10^−9^	46.1	299.2581	−2.54	C_18_H_34_O_3_	95.0851(100)71.0863(82.07)	299.2592
Phosphorylated sphingolipid	4.83×10^−9^	30.0	352.2256	−1.55	C_16_H_34_NO_5_P	236.2366(100.0)184.0694(25.99)	352.2247
Sterol lipid	1.44×10^−8^	45.5	447.3470	−2.19	C_28_H_46_O_4_	429.3376(43.06)411.3276(32.91)	447.3470
Protein	2.05×10^−8^	33.3	966.5938	−3.09	-		
Protein	5.24×10^−8^	31.7	1086.2922	−2.76	-		

a Fragments collected under a collision-induced dissociation energy of 20 eV.

b FTMS accurate mass was not obtained for this compound. This formula is based on TOF-MS mass accuracy.

Statistical values such as p-value and fold change were determined by XCMS analysis of the *O. volvulus* + and *O. volvulus* − mass spectral data files. Retention time (RT), and mass to charge value (*m/z*), fold change and the direction of overall ion intensity change, represents the average value across all files. Molecular formula and compound class identifier as determined by MS/MS and FTMS analysis is provided.

### Multivariate analysis of African serum and plasma biomarkers

**Figure 2:**
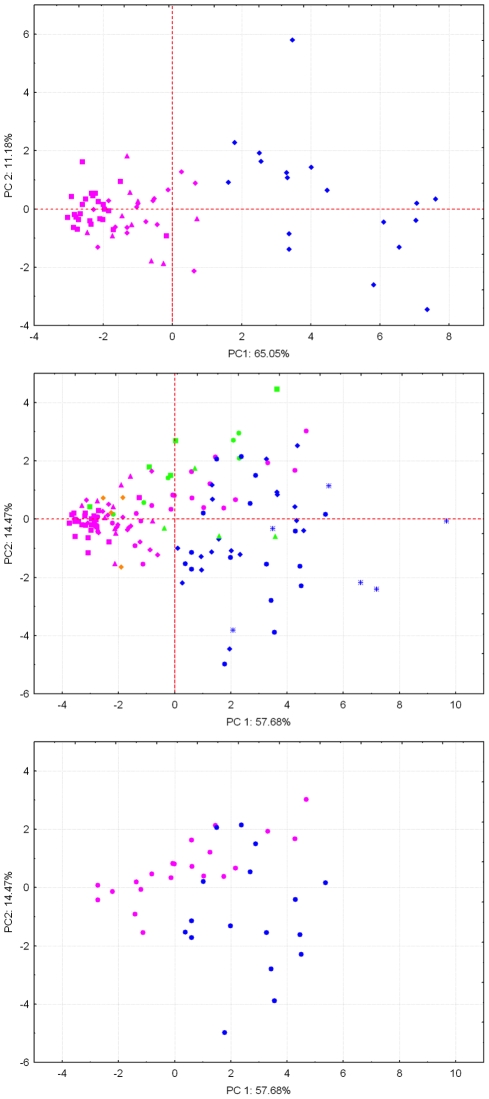
PCA factor score plots of MS peak intensity values for the 14 candidate onchocerciasis biomarkers. Mass feature intensity values were extracted through ESI-TOF+/XCMS analysis of (A) African blood serum and plasma samples from 55 *O. volvulus* infected individuals compared against 18 healthy controls. (B) A sample set including 76 *O. volvulus* infected individuals compared against 56 *O. volvulus* negative controls (including healthy and those infected with other tropical diseases). (C) An extraction of only those data points representing the 21 Guatemala *O. volvulus* infected individuals compared against 18 healthy controls. Individual data points are symbolized using the following code for country of origin and disease status: “blue diamond” = Cameroon *Ov*−, “blue circle” = Guatemala *Ov*−, “blue astrisk” = Scripps *Ov*−, “green circle” = Leishmaniasis *Ov*−, “green square” = Chagas *Ov*−, “green triangle” = LF *Ov*−, “pink diamond” = Cameroon *Ov*+, “pink circle” = Guatemala *Ov*+, “pink square” = Ghana *Ov*+ (1986, 1991 and 2003 samples), “pink triangle” = Liberia *Ov*+, “orange diamond” = Cameroon *Ov*?.

Beginning with a subset of the larger sample set, the mass spectral data for the top 14 candidate biomarkers were investigated for their ability to discriminate *O. volvulus* infected individuals (n = 55) from healthy controls (n = 18) from the African serum and plasma samples. PCA of the effect of these 14 biomarkers was used to visualize the variation between these samples groups (Figure 2A). A distinct clustering of the *O. volvulus* infected versus the healthy individuals was observed across the x-axis of the PCA score plot, implying that principal component 1 (PC1) contained the variance of the data set required to distinguish these two sample groups. The next greatest amount of variation within the data set appeared to have little effect on discriminating infection or even geographic differences, but appears to be more representative of the heterogeneity present among healthy controls.

### Multi-region multivariate analysis

The top 14 candidate biomarkers were also applied to a larger sample set comprised of multiple geographic regions including *O. volvulus*-infected individuals (n = 76) and healthy and disease controls (n = 56). PCA of these 14 biomarkers (Figure 2B) revealed the inherent complexity encountered when employing a metabolite profiling approach to diagnostic development. As with the initial African samples, there is general clustering of the onchocerciasis positive individuals with the variance contained in PC1 having good discriminatory power. The disease and healthy controls cluster separately from the onchocerciasis positive individuals, however, there is some overlap between one of the Chagas disease and two of the leishmaniasis positive individuals. Interestingly, the lymphatic filariasis samples, infected with the closely related filarial parasite *Wuchereria bancrofti*, cleanly cluster with the healthy controls.

Ideally serum and plasma samples would not be directly compared against each other as the two matrices have distinct chromatographic differences (Figure S2). However, given the nature of onchocerciasis sample banks that have been collected over the past 20 years, it was important to determine if the resulting biomarker results would be biased to one biological sample type over another. Importantly, our results show that the plasma samples from Cameroon as well as the Indian lymphatic filariasis plasma samples consistently align as expected with the multi-region serum sample set in distinguishing onchocerciasis infected from uninfected individuals.

### Guatemala central endemic zone metabolic signature variability

As evidenced in Figure 2C, there is little clustering of the Guatemalan individuals initially classified as onchocerciasis positive; rather there appears to be a continuum of onchocerciasis disease variation within those samples. However, dissections of excised nodules at the time of nodulectomy revealed no live worms, as opposed to the results of the Cameroon samples where infection status was confirmed by the extraction of live *O. volvulus* worms.

### Machine learning algorithm implementation

Although tools such as PCA provide a graphical means of distinguishing between sample groups, they do not have the ability to provide a quantitative diagnostic assessment as would be needed nor are they intended to be used for field applications of an onchocerciasis diagnostic. Alternatively, machine learning algorithms do provide the necessary binary output, as well as calculate confidence intervals of a given classification. The mass spectral intensity values for the onchocerciasis serum and plasma data set were used as inputs in a collection of machine learning algorithms. The algorithms were chosen to provide a survey of the various types of machine learning algorithms that could be used with mass spectral data in diagnostic assessments, either alone or in combination in more sophisticated algorithms. Results of this analysis are summarized in Table 3 where sensitivity (true positive rate) and specificity (1–false positive rate) are displayed. The receiver operating characteristic (ROC) areas present a numerical value description of the relationship between sensitivity and specificity for a given diagnostic test [35], [36]. In the context of a binary classification problem as presented here, a value of 0.5 indicates there is no discrimination within the test and shows any result is essentially the same as a random guess, while a value of 1.0 indicates a perfect test prediction. Based upon the data, it is clear that the inclusion of the Guatemala samples within the sample analysis dramatically increases the number of reported false positives, compromising the accuracy of the test overall. However, it is important to note that within the context of the Africa sample set, the ROC area approaches, or is equal to, a perfect test prediction in numerous cases, and in the case of the functional trees classification tree algorithm, perfect sensitivity and specificity can be achieved.

**Table 3:** Summary of the diagnostic accuracy of the machine learning algorithm analysis.

Algorithm	Classifier type	Entire sample set			Africa samples		
		Sensitivity	Specificity	ROC area	Sensitivity	Specificity	ROC area
BayesNet	Bayesian network	84.8	87	0.929	97.3	99.1	1
NaiveBayes	Bayesian network	88.6	88.3	0.930	94.5	98.2	1
Logistic	Logistic regression	85.6	85.2	0.898	98.6	99.6	0.999
IB1	Nearest neighbor	87.1	83.9	0.855	98.6	95.8	0.972
OneR	Minimum error attribute	76.5	76.6	0.766	89.0	85.2	0.871
Multilayer perceptron	Backpropagation classification	87.9	85.9	0.921	98.6	95.8	1
FLR	Fuzzy lattice reasoning	81.1	83.7	0.824	98.6	99.6	0.991
Functional trees	Classification tree	84.1	83.6	0.861	100	100	1
Random forest	Classification tree	88.6	89.3	0.954	97.3	95.4	0.997

The mass spectral ion intensities of the top 14 candidate onchocerciasis biomarkers from onchocerciasis infected and uninfected samples were compared between the multi-region sample set and the African blood samples. All results were obtained using a 10 fold cross validation analysis.

## Discussion

Metabolomics, or the measurement of all the metabolites present in an organism, and metabolite profiling, in which a smaller subset of metabolites are measured, have become established as useful tools in the “real-time” measurement of organismal metabolism. For infectious disease, previous metabolomics approaches have included mice challenged with the protozoan parasites *Trypanosoma brucei brucei* [37] and *Plasmodium berghei* [38], trematode parasites *Echinostoma caproni* [39] and *Schistosoma mansoni* [40] and some viruses [41]. This study represents the first investigation of a metabolomic approach to the discovery of biomarkers and creation of a diagnostic test for identifying and classifying onchocerciasis infection. Through the use of multivariate statistics and machine learning algorithms, the potential of metabolomic analysis has been demonstrated for uncovering biomarkers for specific determination of not only onchocerciasis infection but holds promise for the diagnosis of other parasitic diseases. Specifically, this was demonstrated by the clustering of the *W. bancrofti* infected samples with those individuals that were not infected with *O. volvulus* in the multivariate PCA. This clustering showed the potential specificity of the biomarkers for the discrimination of onchocerciasis from other filarial diseases. Although this analysis consists of only four representative lymphatic filariasis samples, the distinct clustering of these samples with uninfected individuals is noteworthy and argues for future analysis that includes other filarial disease pathogens (e.g., *Brugia malayi*, *Loa loa*).

The 14 candidate biomarkers showed excellent performance in the African specific sample set with up to 99–100% sensitivity and specificity when examined with the single machine learning algorithms. With 99% of onchocerciasis disease prevalence in Africa [42] and the presence of multiple regions of ongoing transmission [43], this is the most clear test of the biomarker strategy.

When applied to a multi-region sample set, the multivariate PCA of the biomarker analysis resulted in a wide spread of results across the range of infected and uninfected individuals. This observation raises several questions regarding the unique epidemiological challenges of measuring onchocerciasis in the Americas. In the context of the PCA, the Guatemalan patients did not classify as expected if nodule presence alone is used as an indicator of infection. However, nodule presence as a diagnostic is known to have exceedingly poor sensitivity and specificity. A possible explanation of this data is that the observed heterogeneity is related to microfilarial load. Unfortunately, skin snip samples with mf counts were not collected for the Guatemala sample set. Nonetheless, if this observed spread of data were correlated with variation in the presence of the mf, then in a region such as the Guatemalan Central Endemic Zone (CEZ) where biannual dosage of ivermectin reaches high coverage levels [44], mf should be nearly absent and we would expect to see no spread of the data but rather a distinct cluster with or near the uninfected individuals. Alternatively, the observation that a quarter of the nodules from these infected individuals from the Guatemalan CEZ did not contain living worms, indicates that these biomarkers may be sensitive to not only the presence, but also the viability of the infective worms. The results of this PCA are consistent with an increasing body of evidence that biannual ivermectin treatments, as are received in the Guatemalan CEZ, have an effect on the viability of adult female worms and ultimately on the elimination of parasites [45]–[47]. Since the Guatemalan *O. volvulus* positive samples do not segregate along clear lines with the clinically confirmed samples from Africa, it is possible that the continuum seen in the PCA plot reflects a range of infection that could be correlated qualitatively or quantitatively to the health of the worms (e.g., live healthy, dying, and dead) *in vivo*. Given that an individual with dead or dying worms does not need further treatment in the context of ivermectin mass drug administration, this finding is particularly valuable in the context of onchocerciasis elimination progress. Ideally, a biomarker determination study would involve independent sample sets for training, validation, and testing. Due to sample limitations inherent to onchocerciasis and many neglected tropical diseases in general, we have chosen to use an approach that trains on the majority of the sample set, and through the 10-fold cross validation machine learning analyses, conduct tests on small subsets of the full sample set [48].

In this study, we report only those features detected in positive ion mode with the highest statistical significance and the most accurate intensity values by XCMS analysis. Consistent among these 10 small molecule features is that they are all fatty acids and related fatty acid derivatives. Further investigations into the biological roles of these fatty acids and fatty acid sterols in onchocerciasis disease progression and potential interaction with the down-regulated proteins is of distinct interest, not only in the development of a diagnostic but also to more clearly understand the biology of this disease. Almost certainly, other biomarkers could be discovered and validated simply by altering the chromatographic (e.g., HILIC) and/or ionization conditions (e.g., negative mode ESI, APCI). It is possible that additional markers can be eventually be added to the repertoire of biomarkers used for onchocerciasis detection, further increasing assay specificity.

The achievement of the goals of elimination and eradication of onchocerciasis and of the neglected tropical diseases in general, ultimately depends upon the ability to measure and track the progress of disease elimination and recrudescence. Our study highlights advantages of a metabolomics based diagnostic over onchocerciasis diagnostics currently implemented including: sensitivity, reproducibility, invasiveness, and the potential for multiplexing with biomarkers for other filarial and/or neglected tropical diseases. Fine calibration of this test in the Western Hemisphere would require characterized samples from individuals with confirmed active infection. Unfortunately, these samples are rapidly becoming a rarity due to the success that has been achieved by OEPA. Further refinement and validation of this metabolomic based diagnostic approach calls for an expansion of the mass spectral analysis with larger sample sets, while inclusion of a greater demographic representation will allow for further validation of the test in specific populations (e.g., children, adults, different genetic backgrounds). Eventually, the optimized biomarkers can be ported into field-based technologies (e.g., immuno-chromatographic or micro-fluidic-based tests) for use as a point-of-care diagnostic, a determinant for the distribution and duration of treatment, and ultimately for long-term disease surveillance.

## Supporting Information

Figure S1 Extracted Ion Chromatograms of the 14 candidate biomarkers as determined from XCMS analysis of *O. volvulus* +(−) and *O. volvulus* −(−) mass spectral data files.
(0.56 MB TIF)

Figure S2 An overlay of representative serum (−) and plasma (−) TICs (total ion chromatogram) collected from TSRI normal blood.
(0.05 MB TIF)
