# Supplementary figures and images for: Metabolomics-Based Discovery of Diagnostic Biomarkers for Onchocerciasis
Source: PLoS Negl Trop Dis. 2010 Oct 5;4(10):e834. doi: 10.1371/journal.pntd.0000834 (PMC2950146; doi:10.1371/journal.pntd.0000834)

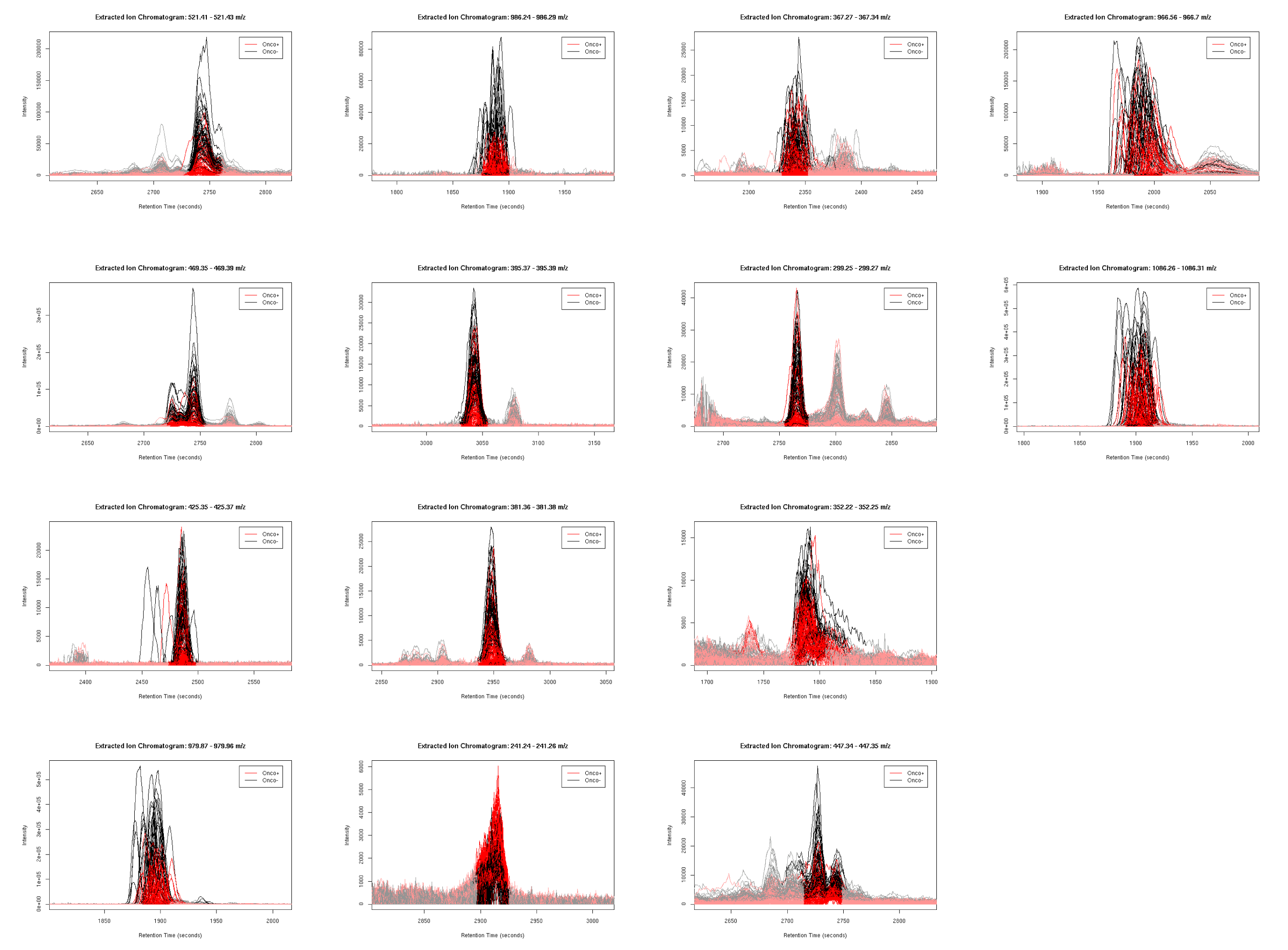

Supplement: Figure S1 — Extracted Ion Chromatograms of the 14 candidate biomarkers as determined from XCMS analysis of O. volvulus +(−) and O. volvulus −(−) mass spectral data files. (0.56 MB TIF) [file pntd.0000834.s001.tif]

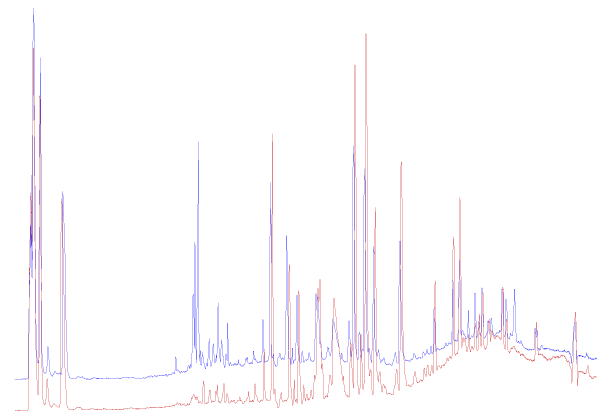

Supplement: Figure S2 — An overlay of representative serum (−) and plasma (−) TICs (total ion chromatogram) collected from TSRI normal blood. (0.05 MB TIF) [file pntd.0000834.s002.tif]
